# From automated scoring to learning diagnosis: a mechanism study of AI-supported formative assessment in English writing

**DOI:** 10.3389/fpsyg.2026.1905455

**Published:** 2026-09-23

**Authors:** Xiao-Chao Yao, Lin Fan

**Affiliations:** Foreign Language Teaching Department, Hainan Vocational University of Science and Technology, Haikou, Hainan, China

**Keywords:** AI-supported formative assessment, automated written feedback, English writing, feedback literacy, learning diagnosis, revision quality, self-regulated learning, teacher review

## Abstract

**Introduction:**

AI-supported writing assessment can provide rapid scores and issue-level comments, yet its formative value depends on how students and teachers interpret and act on that information. This study examined this process in a university English writing course.

**Methods:**

Two pre-existing intact classes comprising 96 students (48 per class) completed three writing-feedback-revision cycles. The Control class received AI scores/standard feedback, routine teacher checking, and revision. The Diagnostic Review class received DeepSeek-R1 issue-level diagnoses, completed an interpretation sheet, revised independently, underwent structured teacher review and reassessment, and recorded a reflection. Measures included diagnostic accuracy, feedback actionability, feedback comprehension, self-assessment accuracy, revision quality, reflection quality, misunderstanding codes, and teacher review activity. Mixed-effects models with student random intercepts were used for analysis.

**Results:**

The Diagnostic Review class showed higher scores for diagnostic accuracy, actionability, comprehension, revision quality, and self-assessment accuracy. Group-by-cycle interactions were significant for diagnostic accuracy, comprehension, revision quality, and self-assessment accuracy, whereas the actionability interaction was not. Feedback comprehension showed the strongest bivariate association with revision quality. Exploratory indirect associations through actionability and comprehension remained positive after adjustment for condition and cycle using student-cluster bootstrap confidence intervals. Teacher records documented confirmation, correction, supplementation, and re-explanation, with greater review time in the Diagnostic Review class.

**Discussion:**

The findings indicate that the formative value of AI-supported writing assessment extends beyond automated diagnosis to the subsequent processes of student interpretation, revision, and teacher review. Repeated classroom assessment may therefore benefit from integrating AI-generated diagnostic feedback with structured learner reflection and context-sensitive teacher judgment.

## 1 Introduction

Formative feedback supports L2 writers when it helps them compare a draft with task standards, identify a gap, and choose a workable revision move (Sadler, 1989; Black and Wiliam, 1998; Hattie and Timperley, 2007; Shute, 2008). In writing classrooms, comments become useful through student activity: learners locate the problem, interpret its relevance, judge its priority, and alter the text. Research on written feedback has consequently moved from delivery to uptake and textual change (Hyland and Hyland, 2006); feedback-literacy research describes the interpretive and evaluative capacities that make uptake possible (Carless and Boud, 2018; Winstone et al., 2017). These demands are substantial when feedback concerns grammar and vocabulary alongside coherence, evidence, organization, and argument development, all of which require different kinds of judgment.

Automated writing evaluation addresses part of this problem by increasing the speed and density of available feedback. Earlier studies concentrated on score reliability, efficiency, and agreement with human ratings (Warschauer and Ware, 2006; Dikli and Bleyle, 2014); later work examined comments on grammar, vocabulary, organization, readability, and revision (Li et al., 2015; Stevenson and Phakiti, 2014; Ranalli, 2018). Generative systems now produce fluent, extended explanations and have prompted research on engagement, digital literacy, and revision behavior (Zhang and Hyland, 2018, 2025; Yeung, 2025). Review evidence also highlights implementation and alignment challenges (Abudalfa and Barrot, 2026). The instructional question now concerns how learners interpret diagnostic output, how teachers check it, and how both activities shape revision.

For formative assessment, a score and an issue-level diagnosis provide different kinds of evidence. The diagnosis specifies a problem, its location and severity, and a possible revision direction; students still have to decide whether the comment fits the task and how to act on it. Feedback-literacy and feedback-ecology studies place this interpretation within criteria, timing, classroom participation, and teacher mediation (Carless and Boud, 2018; Winstone et al., 2017; Tam, 2024). Intervention studies show that restating comments, identifying uncertainty, and planning a revision can strengthen these capacities (Little et al., 2024; Lei and Lei, 2026). Teacher-feedback research further describes the work of confirming, correcting, supplementing, and re-explaining automated diagnoses, especially for discourse-level problems (Wu et al., 2023; Xu et al., 2024; Lee et al., 2023; Lee and Mao, 2024). What remains less clear is how these elements co-occur across repeated writing cycles and how they relate to revision quality and self-assessment.

We examined a diagnostic-review routine in one university English writing course. Two intact classes completed the same tasks and rubric across three cycles. The Control class received AI score/standard feedback, routine teacher checking, and revision. The Diagnostic Review class received DeepSeek-R1 issue-level diagnosis, interpreted the comments before revising, received structured teacher review after revision, underwent reassessment, and completed a reflection. The study asked: **RQ1**. How closely did issue-level AI diagnoses align with teacher-confirmed diagnoses across issue types and writing cycles? **RQ2**. How were diagnostic accuracy, feedback actionability, and feedback comprehension related to revision quality? **RQ3**. How did self-assessment accuracy and writing performance differ between conditions across cycles? **RQ4**. What teacher review actions and workload were recorded around AI-supported feedback use? H1 predicted higher comprehension, revision quality, and writing gains in the Diagnostic Review class. H2 specified exploratory indirect associations from diagnostic accuracy to revision quality through actionability and comprehension.

## 2 Related work

### 2.1 AI-supported written feedback and automated scoring in English writing

Automated scoring research established reliability, efficiency, and human-machine agreement as central assessment questions (Warschauer and Ware, 2006; Dikli and Bleyle, 2014). Classroom studies then examined how learners used comments on grammar, vocabulary, organization, readability, and revision (Li et al., 2015; Stevenson and Phakiti, 2014; Ranalli, 2018). The evidence shows that automated feedback can make support more immediate, while uptake varies with the type of problem and the learner's ability to interpret the comment. Local language issues are easier to locate and act on; discourse-level issues require a view of the task, the paragraph, and the argument.

Generative AI extends this line of work by producing fluent explanations and multiple revision options. Recent studies connect AI-supported feedback with engagement, digital literacy, self-regulation, and writing performance (Guo et al., 2024; Yeung, 2025; Sari and Han, 2024). Critical scholarship also places AI feedback within questions of authorship, academic integrity, task purpose, and disciplinary expectations (Kubota, 2023; Pecorari, 2023). The central empirical issue is now the organization of feedback use after a draft has been scored.

### 2.2 Formative assessment and diagnostic use of feedback

Formative assessment places feedback within a cycle of evidence, judgment, and action. Standards provide the reference point for judging a draft, while feedback identifies the gap and suggests a next step (Sadler, 1989; Black and Wiliam, 1998; Hattie and Timperley, 2007; Shute, 2008). In writing, an overall score locates performance on a scale; an issue-level diagnosis locates a problem in the text and describes its severity, rationale, and possible revision direction. These forms of evidence become useful when students connect them to the task and when teachers check their fit with the rubric and the draft (Vaughan and Uribe, 2024; Tam, 2024).

AI systems can increase the speed and density of this evidence. Their comments become formative when they remain connected to the writing task, rubric, and classroom decisions. Feedback that names a problem, locates it, explains its effect, and points to a feasible action gives students a basis for revision; teacher checking supplies contextual judgment when the issue concerns meaning, evidence, or rhetorical purpose.

Studies of large language models report rapid explanation and adaptive support, alongside continuing questions about alignment with learning goals (Steinert et al., 2024; Calli and Er, 2026). Diagnostic comments provide the starting evidence for a feedback-use sequence in which students interpret the diagnosis, revise against it, and bring difficult cases to teacher review.

### 2.3 Feedback literacy and student uptake of AI feedback

Feedback literacy concerns the learner's ability to interpret, evaluate, and use comments. Students need to restate what a comment addresses, explain why the issue matters, identify uncertainty, and choose a revision action (Carless and Boud, 2018; Winstone et al., 2017). Intervention studies show that comparing comments with criteria, explaining them in one's own words, and planning a revision can strengthen these capacities (Little et al., 2024; Lei and Lei, 2026).

AI feedback makes this interpretive work especially important because fluent language can obscure variation in accuracy, specificity, and priority. AI feedback literacy research describes uptake as a cycle of understanding, evaluation, and use in which learners retain responsibility for writing decisions (Liu and Deris, 2025). Studies of ChatGPT feedback and metacognitive feedback similarly link engagement to questioning suggestions and connecting them with task goals (Zhan and Yan, 2026; Teng and Ma, 2024; Lu et al., 2024).

Feedback comprehension captures this work more directly than satisfaction ratings. Interpretation sheets, revision plans, and reflection records require learners to state the problem, explain its effect, identify uncertainty, and specify a revision direction. These records place comprehension between diagnostic output and textual change.

### 2.4 Self-regulated learning, self-assessment accuracy, and revision studies

Self-regulated learning connects feedback with goal setting, monitoring, strategy selection, and reflection (Butler and Winne, 1995; Nicol and Macfarlane-Dick, 2006). In AI-supported writing, diagnostic information matters when students compare it with their own judgment, monitor the gap across drafts, and select a revision strategy. Recent generative AI studies link this activity with learner traits, literacies, and motivational conditions (Weng et al., 2024; Anders and Dux Speltz, 2025; Chiu, 2024).

Self-assessment accuracy captures the alignment between a student's judgment and a teacher- or rubric-based criterion. Reviews of self-assessment show that calibration depends on task conditions, criteria, and instructional support (Panadero et al., 2016, 2017; Andrade, 2019). Issue-level diagnosis gives students another reference point for comparing their judgment with external evidence and teacher review (Leon et al., 2023).

Revision research shows that edit counts alone provide a weak account of feedback use. Student response and textual change reveal whether a revision targets the diagnosed problem and strengthens the text (Hyland and Hyland, 2006; Ferris et al., 1997; Zheng and Yu, 2018). Revision quality includes problem targeting, adequacy, depth of change, alignment with feedback, and improvement in meaning or organization.

Teacher feedback research adds the classroom judgment required when automated comments are incomplete or difficult to apply. Teachers confirm diagnoses, correct misplaced or overstated comments, supplement missing guidance, and re-explain priorities in terms students can use (Wu et al., 2023; Lee et al., 2023; Lee and Mao, 2024; Xu et al., 2024; Kim and Hiver, 2025). Review records make these actions and their workload visible alongside self-assessment, revision, and reflection.

Together, these strands leave a classroom-level question: how do issue-level diagnosis, student interpretation, revision action, and teacher review co-occur across repeated writing cycles? The recorded measures cover diagnostic accuracy, student interpretation and revision, self-assessment, reflection, and teacher review.

## 3 Theoretical framework

### 3.1 AI diagnostic feedback as evidence for formative assessment

The classroom sequence connects four established perspectives to distinct forms of feedback activity. Formative assessment explains how task standards and issue-level evidence define a revision need; feedback literacy explains how students interpret and evaluate that evidence; self-regulated learning explains how they monitor the draft and select a revision action; and teacher-feedback research explains the contextual judgment applied during review. Their integration follows the order in which diagnostic information moved through interpretation, revision, reassessment, and reflection.

AI diagnostic feedback contains a problem type, location, severity judgment, explanation, and revision direction. These fields provide issue-level evidence for formative judgment (Sadler, 1989; Black and Wiliam, 1998; Hattie and Timperley, 2007; Shute, 2008). Diagnostic accuracy captures agreement between AI-flagged issues and teacher-confirmed problems; actionability captures the specificity and usability of the information for revision.

Diagnostic accuracy concerns problem identification, location, and severity. Issue-level F1 summarizes agreement while balancing false positives and missed teacher-confirmed issues. Self-assessment accuracy represents the alignment between students' judgments and a teacher- or rubric-based criterion. Issue-level F1 and self-assessment accuracy were calculated using Equation 1:


$$\begin{array}{l} \text{F}1=\frac{2TP}{2TP+FP+FN},\text{ S}{\text{A}}_{ic}=1-\frac{\left|S_{ic}-T_{ic}\right|}{R}. \end{array}$$ (1)

Here *TP* denotes AI-flagged issues confirmed by the teacher, *FP* denotes AI-flagged issues not confirmed by the teacher, and *FN* denotes teacher-confirmed issues missed by AI. *S*_*ic*_ is the student's judgment, *T*_*ic*_ is the teacher or rubric criterion, and *R* is the scale range. Higher values indicate closer agreement.

Feedback actionability is the specificity, contextual clarity, and usability of a comment for revision. Its association with diagnostic accuracy is estimated as:


$$\begin{array}{l} \text{F}{\text{A}}_{ic}=\alpha_{0}+\alpha_{1}\text{D}{\text{A}}_{ic}+\alpha_{2}C_{c}+\alpha_{3}G_{i}+\varepsilon_{ic}. \end{array}$$ (2)

Here FA_*ic*_ is actionability, DA_*ic*_ is diagnostic accuracy, *C*_*c*_ is the cycle factor, *G*_*i*_ is assessment condition, and ε_*ic*_ is the residual.

### 3.2 Feedback comprehension and revision action

Feedback comprehension is the learner's explanation of what a comment addresses, why the issue affects the text, and how the draft should change (Carless and Boud, 2018; Winstone et al., 2017). It is modeled as a function of diagnostic accuracy, actionability, and cycle:


$$\begin{array}{l} \text{F}{\text{C}}_{ic}=\beta_{0}+\beta_{1}\text{D}{\text{A}}_{ic}+\beta_{2}\text{F}{\text{A}}_{ic}+\beta_{3}C_{c}+\beta_{4}G_{i}+\varepsilon_{ic}. \end{array}$$ (3)

Here FC_*ic*_ denotes comprehension; the remaining terms retain the definitions above.

Revision quality combines problem targeting, adequacy, depth of change, alignment with feedback, and improvement in text quality. Its association with the process variables is estimated as:


$$\begin{array}{l} \text{R}{\text{Q}}_{ic}=\gamma_{0}+\gamma_{1}\text{D}{\text{A}}_{ic}+\gamma_{2}\text{F}{\text{A}}_{ic}+\gamma_{3}\text{F}{\text{C}}_{ic}+\gamma_{4}C_{c}+\gamma_{5}G_{i}+\varepsilon_{ic}. \end{array}$$ (4)

Here RQ_*ic*_ denotes revision quality. H2 is examined through the indirect paths from DA_*ic*_ to RQ_*ic*_ through FA_*ic*_ and FC_*ic*_:


$$\begin{array}{l} IE=\left(\alpha_{1}\times \gamma_{2}\right)+\left[\left(\alpha_{1}\beta_{2}+\beta_{1}\right)\times \gamma_{3}\right]. \end{array}$$ (5)

In Equation 5, *IE* is the total indirect association after adjustment for cycle and assessment condition. The terms represent the actionability path and the paths that reach comprehension before revision quality.

### 3.3 Teacher review and the diagnostic-review mechanism

Teacher review comprised confirmation, correction, supplementation, and re-explanation of AI feedback. The teacher checked issue labels against the draft and rubric, added guidance for content or organization, adjusted severity, and translated general suggestions into usable tasks (Wu et al., 2023; Lee et al., 2023; Lee and Mao, 2024; Xu et al., 2024). These activities connect automated evidence with situated writing judgment and make review workload observable.

The classroom sequence was task submission, AI diagnosis, student interpretation, independent revision, structured teacher review, reassessment, and reflection. The Control class received AI score/standard feedback, routine checking, and revision. The Diagnostic Review class received issue-level diagnosis, interpretation prompts, review after revision, reassessment, and an intervention-specific reflection. TR_*ic*_ records confirmation, correction, supplementation, re-explanation, and review time. Because review followed the within-cycle revision, these records describe mediation work around reassessment and reflection.

## 4 Methods

### 4.1 Research context and participants

The study used a quasi-experimental classroom design in a university English writing course. The sample included 96 students from two pre-existing intact classes, with 48 students in each class. One class was assigned to Control and the other to Diagnostic Review at the class level. Both classes followed the same schedule, writing topics, task requirements, rubric, and scoring scale. The three cycles comprised a problem-solution paragraph, a two-paragraph opinion response, and a source-based short response. Control students received AI score/standard feedback, routine teacher checking, and revision. Diagnostic Review students completed the same tasks and the structured diagnostic-review sequence described below.

One course instructor taught both classes and had approximately eight years of university EFL/English-writing teaching experience. The instructor used the same task requirements, rubric, and scoring scale in both conditions, assigned course grades, and completed the teacher review records.

Student records were linked through anonymous identifiers. The analytic file contained 96 participant records and 288 cycle-level observations. Reflection non-submission was coded explicitly. Pretest writing score, feedback literacy, self-regulated learning, and self-assessment accuracy were comparable across classes (Table 1).

**Table 1:** Participant characteristics and baseline equivalence by assessment condition.

Characteristic	Control (*n* = 48)	Diagnostic review (*n* = 48)	*p*-value
Students, *n*	48	48	
Female, *n* (%)	28 (58.3)	31 (64.6)	0.450
Male, *n* (%)	20 (41.7)	16 (33.3)	
Not reported, *n* (%)	0 (0.0)	1 (2.1)	
Age, years	19.15 (0.77)	19.27 (0.87)	0.458
Lower-intermediate, *n* (%)	13 (27.1)	12 (25.0)	0.512
Intermediate, *n* (%)	30 (62.5)	27 (56.2)	
Upper-intermediate, *n* (%)	5 (10.4)	9 (18.8)	
Pretest writing score	66.57 (5.60)	65.56 (6.60)	0.418
Baseline feedback literacy	3.14 (0.38)	3.11 (0.38)	0.706
Baseline self-regulated learning	3.10 (0.35)	3.09 (0.40)	0.890
Baseline self-assessment accuracy	0.71 (0.07)	0.71 (0.09)	0.915

Continuous variables are reported as mean (SD). Categorical variables are reported as n (%).

### 4.2 AI-supported formative assessment mechanism

DeepSeek-R1, accessed through the official web interface, generated diagnostic feedback in all three cycles. The interface did not expose the backend snapshot, and temperature, top-p, and maximum-token settings were not manually configurable. Each prompt contained the task requirements, scoring rubric, and student draft and requested an overall score or level, issue category, problem location, severity, explanation, and actionable revision direction. Diagnosis and revision suggestions were separated, and the model was instructed not to rewrite the composition. Diagnostic Review students completed an interpretation sheet requiring them to restate problems, explain their effects on writing quality, identify uncertainties, and specify a revision plan before independent revision. The same prompt template and rubric fields were used for every student and cycle. One generated output per draft was retained and used in the classroom workflow. The records contain no documented regeneration, selection, researcher editing, or pre-delivery screening.

The instructor reviewed AI feedback and student revision records after each cycle. Review records captured confirmed and corrected AI feedback, added teacher instruction, re-explanation needs, review minutes, review intensity, and review status. Teacher-confirmed issue labels were the reference for AI-teacher diagnostic agreement. Control students received score/standard feedback and routine checking. Diagnostic Review students received issue-level diagnosis, interpretation prompts, structured review after revision, reassessment, and an intervention-specific reflection. The condition-specific workflow across the three writing cycles is summarized in Table 2.

**Table 2:** Condition-specific workflow across the three writing cycles.

Stage	Control	Diagnostic review
Class assignment	One pre-existing intact class (48 students); non-random class-level assignment.	One pre-existing intact class (48 students); non-random class-level assignment.
AI output received by students	AI score/standard feedback. Issue-level AI records were retained for routine teacher checking and coding.	AI score plus issue-level diagnostic feedback with category, location, severity, explanation, and revision direction.
Student interpretation	No structured interpretation sheet. Routine follow-up evidence was available for 135 of 144 student-cycle records (93.8%).	Structured interpretation sheet requiring problem restatement, explanation, uncertainty identification, and a revision plan.
Revision	Revision after score/standard feedback and routine checking.	Independent revision after interpretation of issue-level diagnosis.
Teacher review and reassessment	Routine teacher checking; no intervention-specific reassessment.	Structured teacher review after the within-cycle revision, followed by reassessment.
Reflection	No intervention-specific reflection form; available records are routine course follow-up data.	Intervention-specific reflection after reassessment.
Outcome coding	Same task requirements, rubric, scoring scale, and cycle-level coding framework.	Same task requirements, rubric, scoring scale, and cycle-level coding framework.

The same course instructor taught both conditions.

The fixed prompt was: “You are an English-writing diagnostic assistant. Evaluate the student's draft according to the task requirements and scoring rubric. Provide an overall score/level; identify the main writing problems; assign each problem to an issue category; indicate its location; judge severity; briefly explain why it affects writing quality; and provide an actionable direction for revision. Keep problem diagnosis separate from revision suggestions and do not rewrite the entire composition. Task requirements: [task requirements]. Scoring rubric: [scoring rubric]. Student draft: [draft].”

### 4.3 Measures and coding schemes

The measures were derived from participant records, cycle records, diagnosis counts, revision coding, reflection coding, and teacher review records. Diagnostic accuracy DA_*ic*_ was coded on a 0–1 scale and reflected agreement between AI problem identification, location, severity judgment, and teacher-confirmed diagnoses. Issue-category agreement was summarized with F1 scores using teacher-confirmed labels as the reference.

FA_*ic*_, feedback actionability, was scored on a 1–5 scale for specificity, clarity, and usability. FC_*ic*_, feedback comprehension, was scored on a 1–5 scale for the student's explanation of feedback meaning and intended revision. Diagnostic Review comprehension was coded primarily from interpretation sheets; Control comprehension was coded from available routine follow-up responses and revision evidence using the same rubric. RQ_*ic*_, revision quality, was scored on a 1–5 scale for problem targeting, adequacy, depth of change, feedback alignment, and text improvement. These variables supplied the actionability, comprehension, and revision paths in Equations 2–5.

SA_*ic*_, self-assessment accuracy, was scored on a 0–1 scale as the normalized agreement between student judgment and teacher or rubric criteria. TR_*ic*_, teacher review intensity, was scored on a 1–4 scale from confirmation, correction, supplementation, and re-explanation. Reflection quality was scored on a 1–5 scale. Misunderstanding codes were none, overgeneralized feedback, ignored feedback, accepted AI without judgment, confusion about priority, and not submitted.

Table 3 summarizes the coding ranges. The coding dimensions were aligned with the task rubric and the interpretation, revision, reflection, and teacher-review records; the archived materials do not document a separate validation study of the process coding rubrics. Two independent coders trained and calibrated on approximately 24 records, which were excluded from reliability estimation. They then double-coded 96 of 288 cycle-level records (33.3%), stratified by group and cycle with 16 records per cell. Misunderstanding codes yielded Cohen's κ = 0.82, 95% CI [0.75, 0.88]. The 1–5 ratings for actionability, comprehension, revision quality, and reflection quality yielded ICC(2,1), absolute agreement = 0.88, 95% CI [0.83, 0.92]. The instructor supplied course-level grades and review records; the single-rater grading process has no inter-rater estimate. Formal coder blinding to group membership was not documented.

**Table 3:** Operational definitions and coding scheme for core variables.

Construct	Operational meaning	Scale or coding
Diagnostic accuracy	Agreement with teacher-confirmed problem type, location, and severity.	0–1; higher = greater accuracy.
Feedback actionability	Specificity, clarity, and usability for revision.	1–5; higher = more actionable.
Feedback comprehension	Student explanation of feedback meaning and intended use.	1–5; higher = stronger comprehension.
Self-assessment accuracy	Agreement between student judgment and teacher or rubric criterion.	0–1; higher = greater accuracy.
Revision quality	Targeting, adequacy, depth, feedback alignment, and text improvement.	1–5; higher = higher quality.
Teacher review intensity	Teacher confirmation, correction, supplementation, and re-explanation.	1–4; higher = more intensive review.
Reflection quality	Quality of reflection on feedback interpretation and revision plans.	1–5; higher = higher quality.
Feedback misunderstanding	Misunderstanding or non-use in reflection records.	none; overgeneralized; ignored; accepted without judgment; priority confusion; not submitted.

### 4.4 Procedure and data collection

Data were collected across three writing-feedback-revision cycles. Students completed an initial task, received condition-specific feedback, revised, and submitted follow-up records. Structured teacher review followed the within-cycle revision in the Diagnostic Review condition. Diagnostic Review students completed an interpretation sheet before revision and an intervention-specific reflection after reassessment. Control follow-up records came from routine course monitoring and were available for 135 of 144 student-cycle observations (93.8%); nine observations lacked corresponding follow-up material. The three cycles used paragraph, short-essay, and source-based tasks. The dataset included teacher and AI scores, score gaps, diagnostic accuracy, actionability, comprehension, self-assessment accuracy, revision quality, teacher review intensity, reflection submission, misunderstanding codes, perceived usefulness, feedback trust, learning engagement, issue counts, revision-type counts, and review workload. Identifiers were anonymized before analysis.

### 4.5 Data analysis and validity checks

Baseline variables were compared between groups using descriptive statistics and group tests. Writing improvement was evaluated using gain scores and 95% confidence intervals. Cycle-specific group differences in diagnostic accuracy, feedback actionability, feedback comprehension, self-assessment accuracy, revision quality, and teacher review intensity were summarized using Welch's *t*-tests and Hedges' *g*.

Repeated cycle-level outcomes were analyzed with linear mixed-effects models including Group, Cycle, and Group × Cycle as fixed effects and a student-level random intercept:


$$Y_{ic}=\beta_{0}+\beta_{1}G_{i}+\beta_{2}C_{c}+\beta_{3}\left(G_{i}\times C_{c}\right)+u_{i}+\varepsilon_{ic}.$$

Group had two levels and Cycle had three categorical levels. Estimated marginal means and 95% confidence intervals were calculated by group and cycle. Model checks included variance components, student-cluster Cook's distance, and standardized residuals. Correlations among diagnostic accuracy, feedback actionability, feedback comprehension, and revision quality were calculated. The exploratory indirect-effect analysis included diagnostic accuracy as the predictor, feedback actionability and feedback comprehension as intermediate variables, revision quality as the outcome, and Cycle and Group as covariates. Confidence intervals were estimated from 5,000 bootstrap samples drawn at the student-cluster level. The original cycle-adjusted estimates were retained for comparison. Teacher review intensity, review time, confirmation, correction, supplementation, and re-explanation were summarized by group and cycle. Analyses were conducted in Python 3.13.13 with two-sided α = 0.05.

## 5 Results

### 5.1 Baseline equivalence and writing improvement

The groups were comparable on pretest writing, feedback literacy, self-regulated learning, and self-assessment accuracy (Table 1). Control writing scores increased from 66.57 to 70.15, a mean gain of 3.58 points (SD = 2.26; 95% CI [2.92, 4.24]). Diagnostic Review scores increased from 65.56 to 71.48, a mean gain of 5.92 points (SD = 1.87; 95% CI [5.38, 6.47]). The between-group gain difference was 2.35 points (Hedges' *g* = 1.12, *p* < 0.001). Individual trajectories and gain-score distributions are shown in Figure 1.

**Figure 1:**
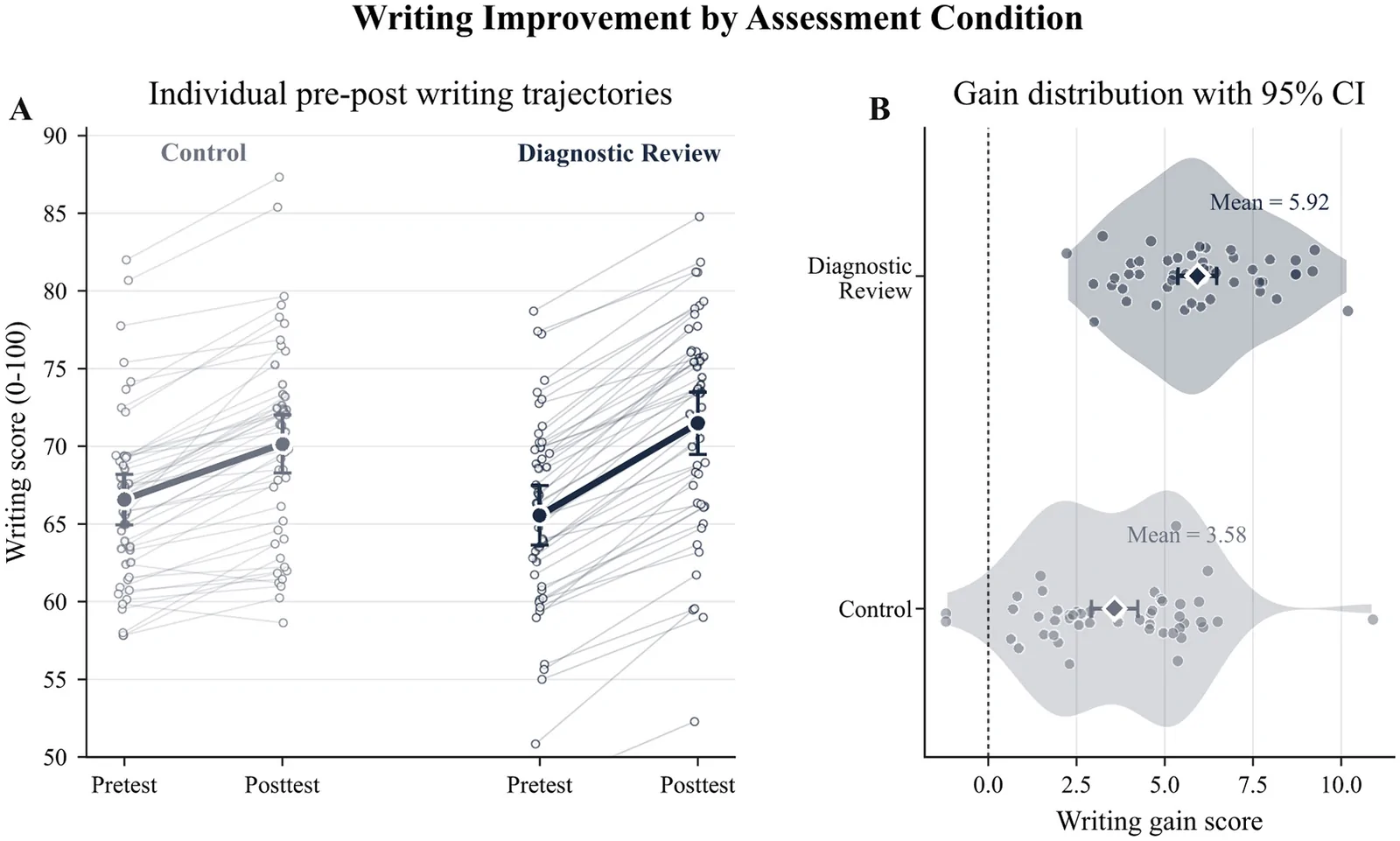
Writing improvement by assessment condition. **(A)** Individual pretest-posttest writing trajectories for students in the Control and Diagnostic Review groups. Thin lines represent student-level paired observations, and large points with error bars indicate group means with 95% confidence intervals. **(B)** Distribution of writing gain scores by group. Violin densities show the full distribution, individual points show student-level gains, and diamond markers with horizontal error bars indicate mean gains with 95% confidence intervals.

### 5.2 Cycle-wise differences in diagnostic and formative assessment indicators

Cycle-level indicators showed clearer group differentiation than the score comparison alone. Diagnostic accuracy was higher in the Diagnostic Review group across all three cycles, with means of 0.696, 0.721, and 0.775, compared with 0.590, 0.619, and 0.619 in the Control group. Standardized group differences were large in each cycle (Hedges' g = 1.35, 1.17, and 1.71; all *p* < 0.001). Feedback actionability and feedback comprehension showed similar patterns. Actionability means in the Diagnostic Review group were 3.42, 3.65, and 3.73 across the three cycles, compared with 2.80, 2.91, and 3.02 in the Control group. Hedges' *g* values ranged from 1.36 to 1.74, with *p* < 0.001 in all cycles. Feedback comprehension means in the Diagnostic Review group were 3.43, 3.68, and 3.83, compared with 3.02, 3.06, and 3.27 in the Control group. Hedges' *g* values were 1.04, 1.33, and 1.36, again with *p* < 0.001 in all cycles.

Self-assessment accuracy showed group differences of *g* = 0.32 (*p* = 0.120), 0.80 (*p* < 0.001), and 1.50 (*p* < 0.001) across Cycles 1–3. Revision-quality differences were *g* = 1.39, 1.50, and 1.86, respectively (all *p* < 0.001). Figure 2 summarizes the cycle-wise effects. Mixed-effects models yielded Group × Cycle interactions for diagnostic accuracy, *F*_(2, 188.4)_ = 3.57, *p* = 0.030; feedback comprehension, *F*_(2, 188.3)_ = 3.18, *p* = 0.044; revision quality, *F*_(2, 188.6)_ = 4.02, *p* = 0.019; and self-assessment accuracy, *F*_(2, 187.9)_ = 10.31, *p* < 0.001. The actionability interaction was not significant, *F*_(2, 188.1)_ = 1.63, *p* = 0.199. Group main effects were significant for diagnostic accuracy, *F*_(1, 94.0)_ = 67.84, *p* < 0.001; actionability, *F*_(1, 94.0)_ = 83.26, *p* < 0.001; feedback comprehension, *F*_(1, 94.0)_ = 51.72, *p* < 0.001; revision quality, *F*_(1, 94.0)_ = 91.43, *p* < 0.001; and self-assessment accuracy, *F*_(1, 94.0)_ = 23.68, *p* < 0.001.

**Figure 2:**
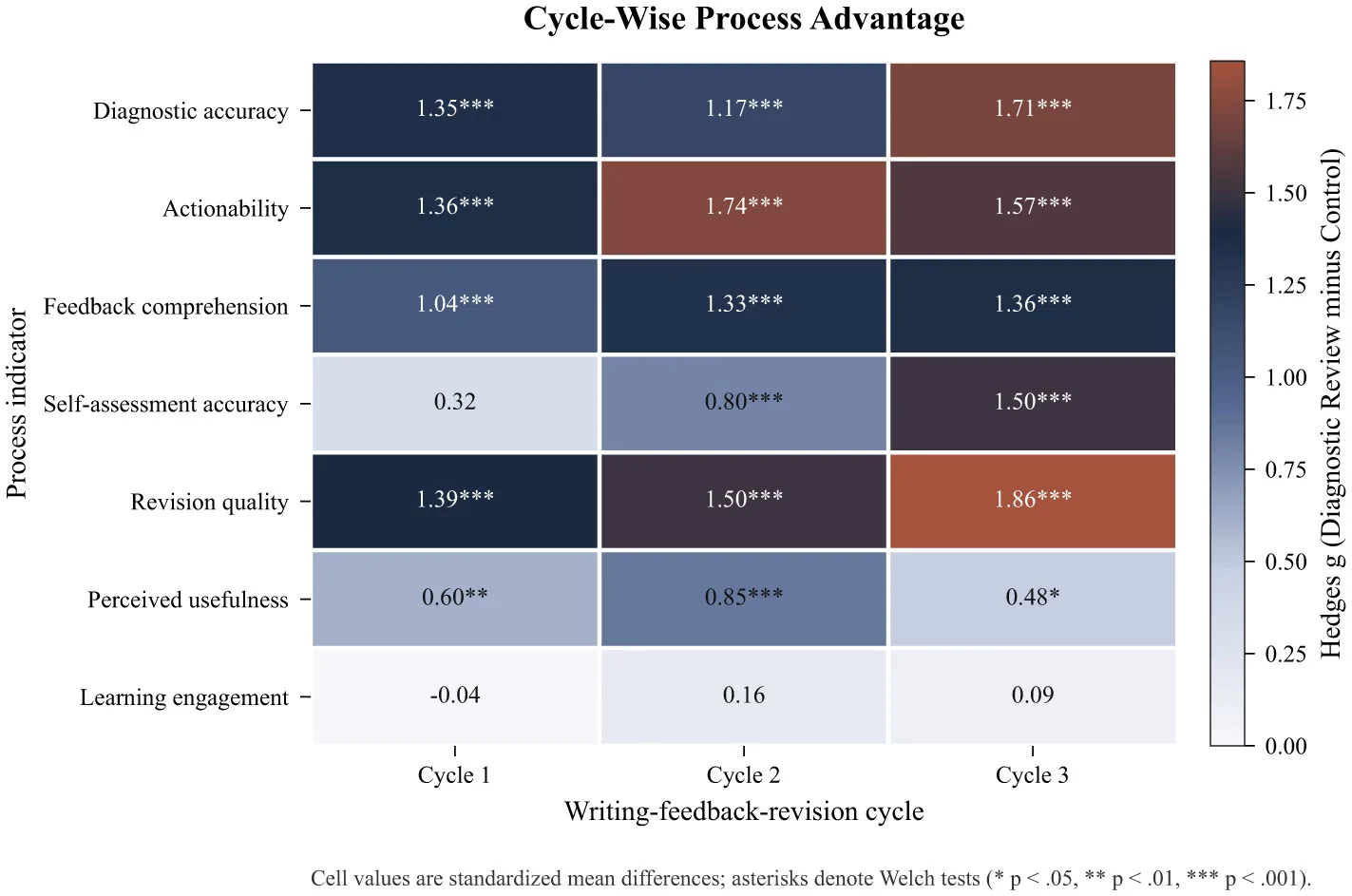
Cycle-wise process advantage across formative assessment indicators. The heat map presents standardized mean differences between the Diagnostic Review and Control groups across three writing-feedback-revision cycles. Cell values represent Hedges' *g*, with positive values indicating higher scores for the Diagnostic Review group. Asterisks indicate Welch's t-test significance levels (*p < 0.05, **p < 0.01, and ***p < 0.001). Darker colors indicate stronger group differences.

Table 4 reports the estimated marginal means. The Diagnostic Review minus Control contrasts for diagnostic accuracy were 0.106, 0.102, and 0.156 across Cycles 1–3. The corresponding contrasts were 0.62, 0.74, and 0.71 for actionability and 0.41, 0.62, and 0.56 for comprehension.

**Table 4:** Estimated marginal means from the repeated-measures mixed-effects models.

Outcome	Cycle	Control EMM (SE), 95% CI	Diagnostic review EMM (SE), 95% CI	Contrast
Diagnostic accuracy	1	0.590 (0.011), [0.568, 0.612]	0.696 (0.011), [0.674, 0.718]	0.106
	2	0.619 (0.013), [0.594, 0.644]	0.721 (0.013), [0.696, 0.746]	0.102
	3	0.619 (0.013), [0.593, 0.645]	0.775 (0.013), [0.749, 0.801]	0.156
Feedback actionability	1	2.80 (0.066), [2.67, 2.93]	3.42 (0.066), [3.29, 3.55]	0.62
	2	2.91 (0.061), [2.79, 3.03]	3.65 (0.061), [3.53, 3.77]	0.74
	3	3.02 (0.065), [2.89, 3.15]	3.73 (0.065), [3.60, 3.86]	0.71
Feedback comprehension	1	3.02 (0.057), [2.91, 3.13]	3.43 (0.057), [3.32, 3.54]	0.41
	2	3.06 (0.067), [2.93, 3.19]	3.68 (0.067), [3.55, 3.81]	0.62
	3	3.27 (0.059), [3.15, 3.39]	3.83 (0.059), [3.71, 3.95]	0.56

Contrast = diagnostic review minus control. SEs for feedback actionability and feedback comprehension are approximate.

The random-intercept and residual variances were 0.09 and 0.11, respectively. The largest student-cluster Cook's *D* was 0.031, and the largest absolute standardized residual was 2.85.

Diagnostic accuracy correlated with revision quality (*r* = 0.445, *p* < 0.001); actionability correlated with revision quality (*r* = 0.480, *p* < 0.001); and comprehension showed the strongest correlation (*r* = 0.501, *p* < 0.001). In the original cycle-adjusted analysis, indirect effects were 0.094 through actionability (95% bootstrap CI [0.040, 0.151]), 0.039 through comprehension (95% CI [0.010, 0.074]), and 0.133 in total (95% CI [0.074, 0.194]). After adjustment for assessment condition with 5,000 student-cluster bootstrap samples, the indirect effects were 0.043 through actionability (95% CI [0.012, 0.078]), 0.024 through comprehension (95% CI [0.005, 0.050]), and 0.067 in total (95% CI [0.024, 0.113]).

### 5.3 AI-teacher diagnostic agreement and teacher mediation

The bubble matrix in Figure 3 presents issue-level AI-teacher agreement. Mean F1 was 0.650 (SD = 0.030) in Control and 0.768 (SD = 0.037) in Diagnostic Review. Diagnostic Review F1 increased from 0.738 in Cycle 1 to 0.803 in Cycle 3. Cycle 3 values were highest for vocabulary word choice (F1 = 0.824), coherence/cohesion (F1 = 0.816), and content/argument (F1 = 0.815); the lowest Control value was source use/evidence in Cycle 1 (F1 = 0.579). Review minutes were 7.82, 8.20, and 8.28 in Control and 10.06, 10.79, and 10.71 in Diagnostic Review across Cycles 1–3. The corresponding Hedges' g values were 0.94, 1.18, and 1.09. Added teacher instruction was 1.27, 1.25, and 1.42 comments per record in Control and 2.60, 2.85, and 3.08 in Diagnostic Review, with *g* = 1.42, 1.50, and 1.61. Review intensity was 1.69, 1.78, and 1.88 in Control and 2.61, 2.75, and 2.94 in Diagnostic Review, with *g* = 2.61, 2.67, and 2.73. Table 5 reports the full review records.

**Figure 3:**
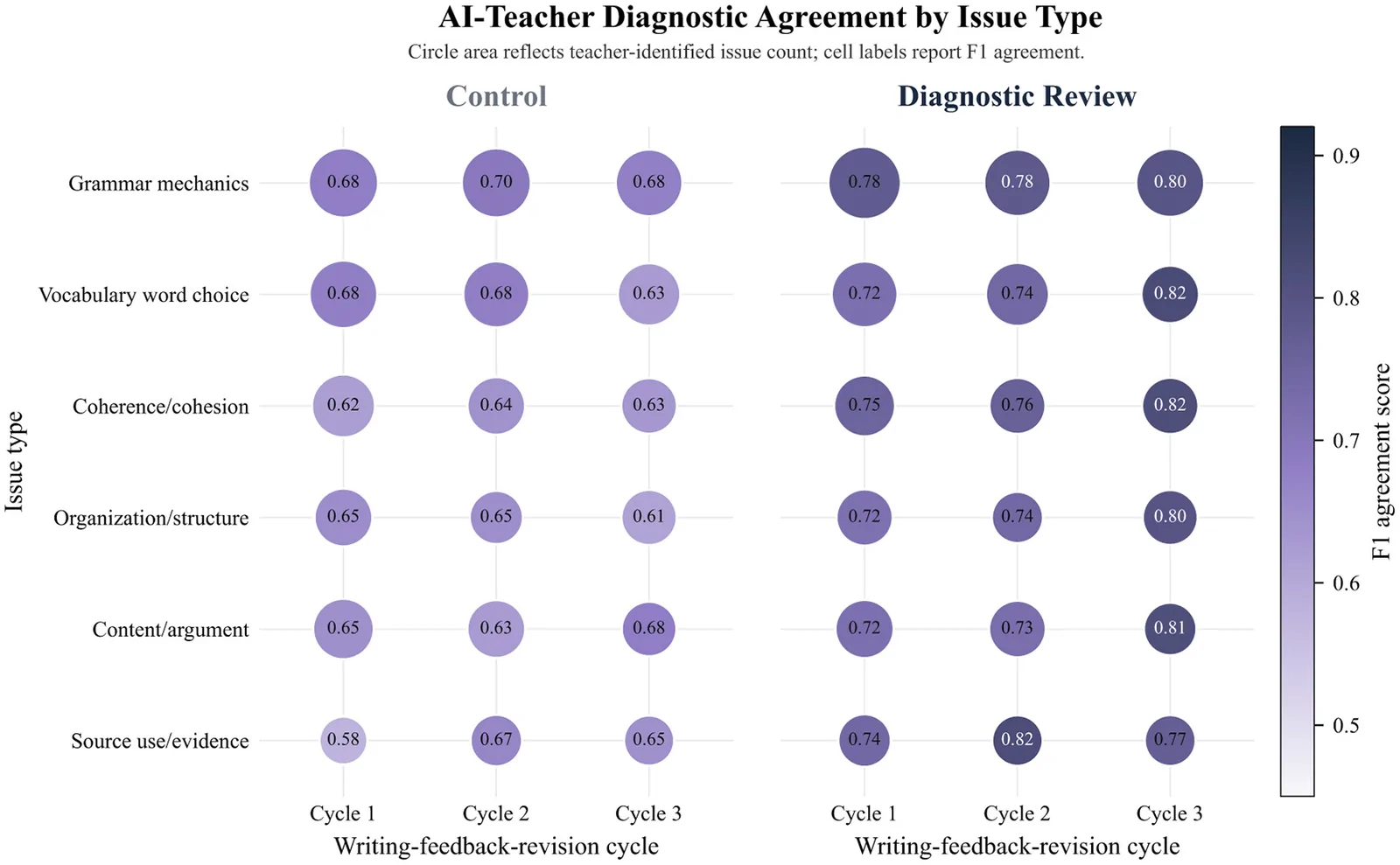
AI-teacher diagnostic agreement by issue type. Bubble matrices compare AI-teacher agreement across issue categories and learning cycles for the Control and Diagnostic Review groups. Circle color indicates the F1 agreement score between AI-flagged issues and teacher-confirmed diagnoses, while circle area reflects the number of teacher-identified issues. Values inside circles report F1 scores for each issue type and cycle.

Table 5Teacher mediation and review workload during AI-supported feedback use.

**Table:** 

Panel A. Review workload and AI-feedback mediation							
Group	Cycle	*n*	Review minutes	Confirmed AI feedback	Corrected AI feedback	Added teacher instruction	Re-explanation needed, *n* (%)
Control	1	48	7.82 (2.54)	3.88 (0.94)	1.79 (0.99)	1.27 (0.87)	14 (29.2)
Control	2	48	8.20 (2.13)	3.90 (1.08)	2.00 (0.99)	1.25 (1.00)	19 (39.6)
Control	3	48	8.28 (2.49)	3.54 (1.09)	1.85 (0.92)	1.42 (0.90)	14 (29.2)
Diagnostic Review	1	48	10.06 (2.20)	4.19 (0.96)	2.40 (1.03)	2.60 (0.98)	13 (27.1)
Diagnostic Review	2	48	10.79 (2.22)	4.21 (1.25)	2.15 (0.95)	2.85 (1.11)	6 (12.5)
Diagnostic Review	3	48	10.71 (1.88)	4.25 (1.02)	2.27 (0.74)	3.08 (1.13)	9 (18.8)

**Table:** 

Panel B. Review status and follow-up records					
Group	Cycle	Teacher review intensity	Completed review, *n* (%)	Brief review, *n* (%)	Follow-up needed, *n* (%)
Control	1	1.69 (0.35)	41 (85.4)	4 (8.3)	3 (6.2)
Control	2	1.78 (0.36)	41 (85.4)	5 (10.4)	2 (4.2)
Control	3	1.88 (0.37)	38 (79.2)	8 (16.7)	2 (4.2)
Diagnostic review	1	2.61 (0.35)	37 (77.1)	9 (18.8)	2 (4.2)
Diagnostic review	2	2.75 (0.36)	41 (85.4)	6 (12.5)	1 (2.1)
Diagnostic review	3	2.94 (0.40)	37 (77.1)	8 (16.7)	3 (6.2)

Continuous variables are reported as mean (SD). Categorical variables are reported as n (%).

### 5.4 Revision architecture and reflective uptake

Revision coding showed higher revision quality in the Diagnostic Review group across all cycles. In Figure 4, the revision-quality line increased from 3.31 to 3.91 in the Diagnostic Review group, compared with 2.70 to 3.09 in the Control group, while the composition of revisions also differed by condition. The Control group remained more dependent on surface-level revision, which accounted for 36.5%, 42.4%, and 40.6% of coded revisions across the three cycles. In the Diagnostic Review group, surface-level revisions stayed near one-third of revisions, while meaning-change, organization, and content/argument revisions formed a larger combined share.

**Figure 4:**
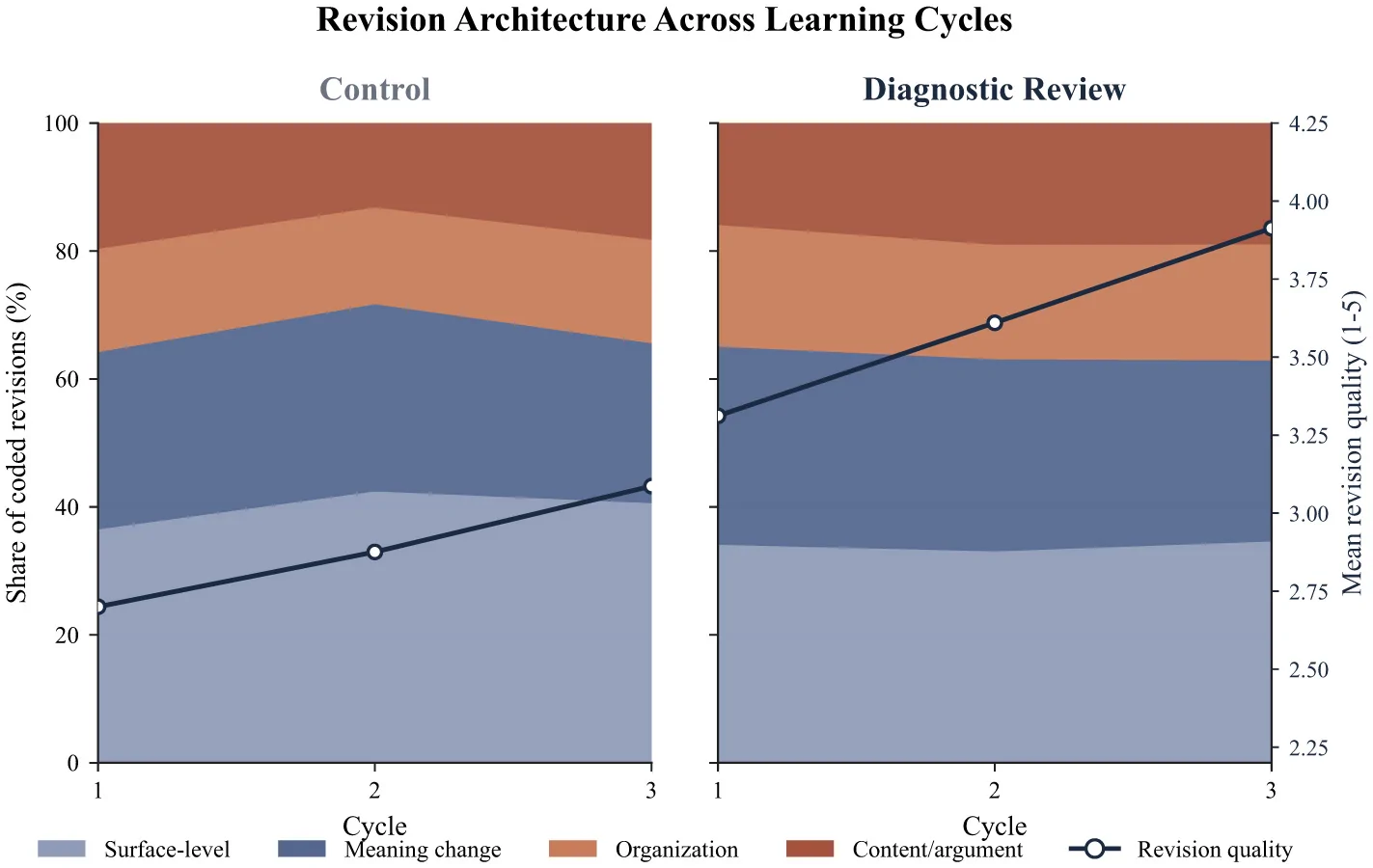
Revision architecture across learning cycles. Stacked area plots show the proportional composition of coded revision types across three cycles, including surface-level revisions, meaning changes, organization revisions, and content/argument revisions. The superimposed line indicates mean revision quality on a 1–5 scale. The figure illustrates how revision behavior and revision quality changed across iterative writing-feedback-revision cycles.

Reflection records contained no coded misunderstanding in 84.0% of Diagnostic Review records and 77.8% of the 135 available Control follow-up records. Control records included more instances of accepting AI without judgment (6.9%) and ignored feedback (4.2%). Diagnostic Review reflection quality was 2.96 (SD = 0.56) in Cycle 1, compared with 2.59 (SD = 0.60) in Control. Monitoring strategy rates ranged from 0.60 to 0.67 in Diagnostic Review and from 0.36 to 0.44 in Control. Figure 5 presents misunderstanding codes and reflection trajectories.

**Figure 5:**
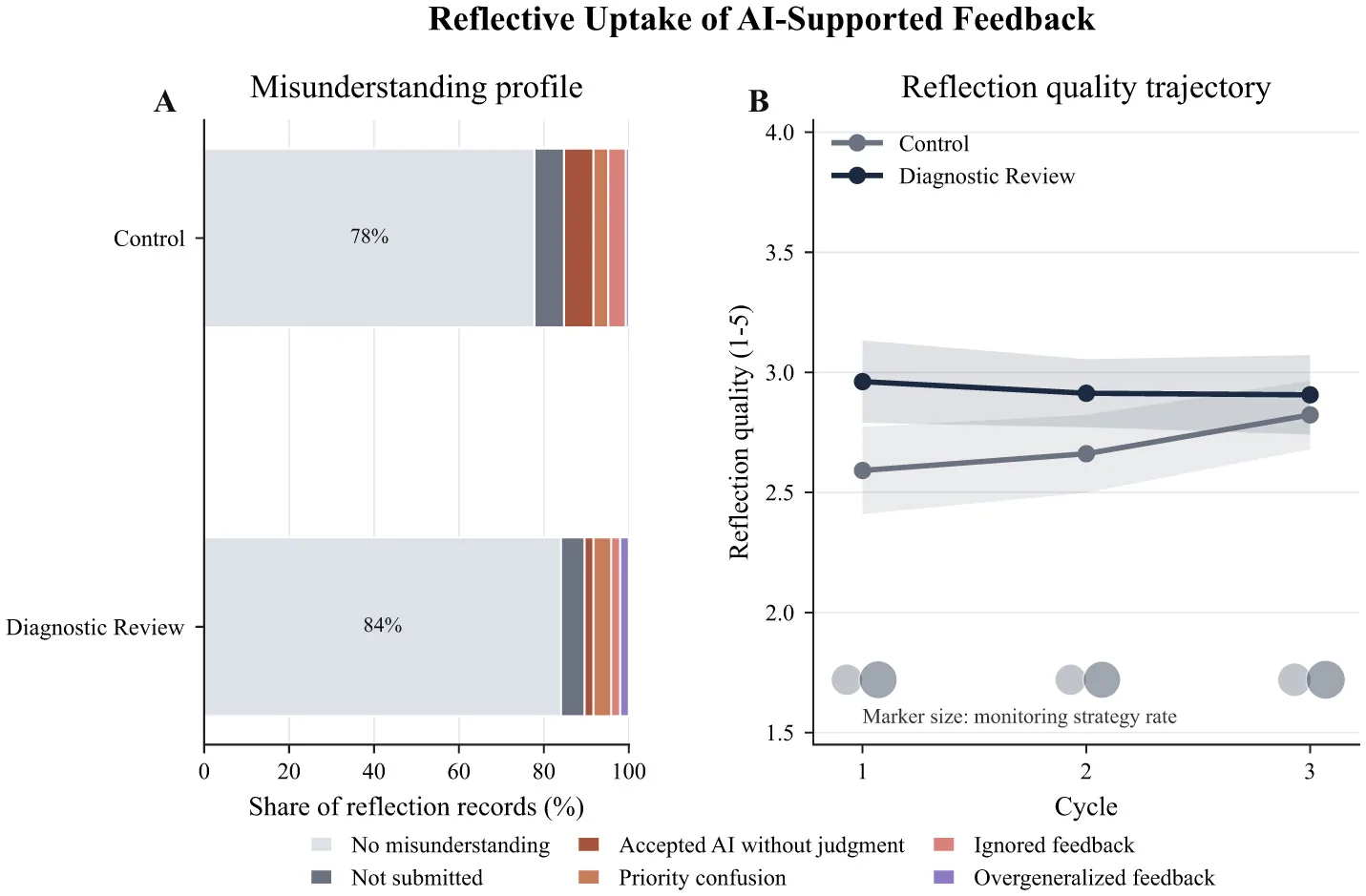
Reflective uptake of AI-supported feedback. **(A)** Distribution of misunderstanding codes in available reflection records by group. Control records were routine course follow-up records (135 of 144 student-cycle observations); Diagnostic Review records were intervention-specific reflections. **(B)** Mean reflection quality across three cycles, with shaded bands indicating 95% confidence intervals. Marker size near the lower axis represents the rate of reported monitoring strategies.

The revision and reflection records show how interpretation accompanied revision architecture. Diagnostic Review revisions contained larger shares of meaning change, organization, and content or argument work, while Control revisions contained a larger share of surface editing. Reflection quality and monitoring strategy rates followed the same group ordering.

## 6 Discussion

### 6.1 Feedback interpretation, revision, and teacher judgment

The Diagnostic Review class gained 5.92 points on writing scores, compared with 3.58 points in Control, and maintained higher diagnostic accuracy, feedback comprehension, and revision quality across the three cycles. Group contrasts varied across cycles for diagnostic accuracy, comprehension, revision quality, and self-assessment accuracy, while actionability remained consistently higher in Diagnostic Review. Comprehension had the strongest association with revision quality. Positive indirect associations linked diagnostic accuracy with revision quality through actionability and comprehension; both remained positive after adjustment for assessment condition and student-level clustering. The pattern places student interpretation at the point where an AI comment becomes a revision decision.

AI-teacher agreement was strongest for issue types with relatively local textual cues. Grammar and vocabulary problems can often be located in a word or sentence. Coherence and organization require relations across sentences and paragraphs, while content and argument depend on task goals and the sufficiency of reasoning. Source use adds a further layer because evidence must function in relation to a claim. The teacher review records reflect these differences through confirmation of clear local diagnoses and correction, supplementation, or re-explanation of context-dependent problems.

The uptake records clarify why comprehension was closely related to revision quality. Diagnostic Review students restated problems, located them in the draft, explained their effects, and set priorities before revising. Their reflections contained fewer instances of uncritical acceptance or ignored feedback, and their revisions included larger shares of meaning change, organization, and content or argument work. Control revisions contained more surface-level editing. These behaviors connect feedback literacy with self-regulated action: comprehension involves selecting an appropriate textual response, not merely recognizing the wording of a comment.

### 6.2 Teacher workload and classroom implementation

Teacher workload increased in the Diagnostic Review class: review time was 10.06–10.79 mins per cycle compared with 7.82–8.28 mins in Control, and added instruction and review intensity were also higher. AI supplied more issue-level information for the teacher to inspect. The teacher's work consequently shifted toward verifying diagnoses, correcting severity, prioritizing issues, supplementing missing guidance, and explaining discourse-level concerns. Interpretation sheets can prepare students to state the issue, its importance, uncertainty, and a revision plan, while teacher time can be directed toward content, organization, coherence, and source use. The observed workload makes implementation planning central to any claim about classroom scalability.

### 6.3 Limitations and future research

The evidence comes from two intact classes taught by one instructor, leaving class selection and instructor effects in the observed group contrast. Student time-on-task was not recorded, and the contrast may include additional interpretation, reflection, and revision time. Feedback comprehension and reflection were derived from structured interpretation and reflection forms in Diagnostic Review and from available routine follow-up and revision records in Control; differences in record source may therefore contribute to the group contrasts on these measures. Teacher learning, confidence, and later unaided feedback practice were not measured. Cycle order coincided with task type, and no delayed post-test was administered. The condition-adjusted student-cluster bootstrap addresses dependence among repeated student records, while the indirect-effect estimates remain exploratory within the intact-class design. Reliability was high for the double-coded process sample [κ = 0.82; ICC(2, 1) = 0.88], while the single-rater course grading process has no inter-rater estimate and formal coder blinding was not documented. The DeepSeek-R1 interface did not expose its backend snapshot or generation settings. Multiclass studies with multiple instructors, teacher-learning measures, student and teacher time measures, factorial comparisons of AI and interpretation activities, and delayed writing assessments would separate these contributions more clearly.

## 7 Conclusion

Across three writing cycles, the Diagnostic Review class showed larger writing gains and higher diagnostic accuracy, feedback comprehension, revision quality, and self-assessment accuracy than the Control class. The process measures trace a sequence in which accurate and actionable comments were interpreted by students and translated into targeted textual change. Teacher records show that AI-supported formative assessment still relies on human judgment, especially for content, organization, coherence, and source use, and that this work requires additional review time. These findings describe how AI diagnosis, student feedback literacy, revision decisions, and teacher mediation operated together in one university course with class-level assignment.

## Data Availability

The raw data supporting the conclusions of this article will be made available by the authors, without undue reservation.
